# Impact of a remifentanil supply shortage on mechanical ventilation in a tertiary care hospital: a retrospective comparison

**DOI:** 10.1186/s13054-018-2198-3

**Published:** 2018-10-26

**Authors:** Daniel A Klaus, Albert M de Bettignies, Rudolf Seemann, Claus G Krenn, Georg A Roth

**Affiliations:** 1 Department of Anaesthesiology, General Intensive Care and Pain Medicine, Medical University of Vienna, Waehringer Gürtel 18-20, A-1090 Vienna, Austria; 2 RAIC Laboratory 13C1, Medical University of Vienna, Waehringer Gürtel 18-20, A-1090 Vienna, Austria; 3 Department of Craniomaxillofacial and Oral Surgery, Medical University of Vienna, Waehringer Gürtel 18-20, A-1090 Vienna, Austria; 4 Department of Anaesthesia and Intensive Care Medicine, Franziskus Hospital, Nikolsdorfergasse 32, A-1050 Vienna, Austria

**Keywords:** Intravenous anesthetics, Opioids, Hospital supplies, Mechanical ventilation, Mechanical ventilator weaning

## Abstract

**Background:**

The continuous administration of opioids in critical care patients is a common therapy for the tolerance of mechanical ventilation. Opioid choice has a crucial impact on the length of mechanical ventilation. Owing to its very short context-sensitive half-life, remifentanil widens the available options for sedoanalgetic strategies. Supply disruption of such established intensive care medication has been reported to worsen clinical outcomes.

**Methods:**

This retrospective study investigated the influence of a nationwide supply shortage of remifentanil on mechanical ventilation and ventilation-associated outcomes at three perioperative intensive care units (ICUs) in a tertiary care hospital in Vienna. Two groups were followed: patients admitted to the ICU during the remifentanil shortage (July 1, 2016 to September 30, 2016) and a control group one year after the remifentanil shortage (July 1, 2017 to September 30, 2017). Included patients were adults, received mechanical ventilation for at least 6 h, were admitted less than 90 days in the respective ICU, and survived their admission.

**Results:**

For comparison, Poisson count regression models and logistic regression models were computed. To compensate for multiple testing, the significance level was split (0.02 for the primary and 0.006 for secondary outcome parameters). Patients in the remifentanil shortage group received significantly longer mechanical ventilation (risk ratio 2.19, 95% confidence interval 2.14–2.24, *P* <0.001) with significantly prolonged ICU stay (*P* <0.001), days with non-invasive ventilation (*P* <0.001), and length of hospital stay (*P* <0.001). No significant difference was found in the occurrence of pneumonia (*P* = 0.040) and sepsis (*P* = 0.061). A greater proportion of patients in the shortage group underwent secondary tracheostomy (*P* <0.001).

**Conclusions:**

The remifentanil shortage caused a significant impairment of essential outcome parameters in the ICU.

**Electronic supplementary material:**

The online version of this article (10.1186/s13054-018-2198-3) contains supplementary material, which is available to authorized users.

## Background

Sedoanalgesia is an essential intravenous treatment in intensive care units (ICUs) and provides tolerance of mechanical ventilation, analgesia, anxiolysis, and reduction of agitation [1, 2]. Opioids are a major component of sedoanalgetic therapies [3]. Side effects of opioid usage in ICUs include prolonged ventilation, psychotropic effects like dysphoria or hallucinations, obstipation, and urinary retention [4]. Prolonged mechanical ventilation with opioid use may result in an increased length of stay in ICUs because of pulmonary complications [5, 6].

The context-sensitive half-life increases for most opioids with increasing duration of infusion. Commonly used opioids approved for intensive care sedation include remifentanil, sufentanil, morphine, hydromorphone, and fentanyl [7]. Remifentanil is a methyl ester that exhibits a fast extrahepatic metabolism determined by non-specific esterases. This non-saturable process results in a high clearance and very brief, non-cumulating half-life of about 10–20 min, which is irrespective of the duration of infusion and as such the only opioid with this property [8].

Between July and September 2016, the General Hospital of Vienna was faced with a nationwide supply disruption of remifentanil, which was the first-line opioid administrated for sedoanalgesia at the ICU. Consequently, physicians had a restricted choice of opioid alternatives for sedoanalgesia performance. Studies indicate that patients receiving a sedoanalgesia regimen with short-acting opioids like remifentanil exhibit a significantly shorter length of invasive ventilation as well as decreased days of ICU stay [9–11].

The aim of this retrospective study was to investigate the potential influence of the supply disruption of remifentanil on the length of mechanical ventilation during the supply disruption as compared with patients admitted to the ICU during a comparable time period with no opioid restrictions. We hypothesized that ICU patients exhibit prolonged ventilation time because of a coerced shift in opioid choice. Furthermore, we investigated potential differences of the length of ICU stay, duration of non-invasive ventilation, length of hospital stay, and occurrence of pneumonia and sepsis during the two time periods.

## Methods

### Patient data acquisition

**Fig. 1:**
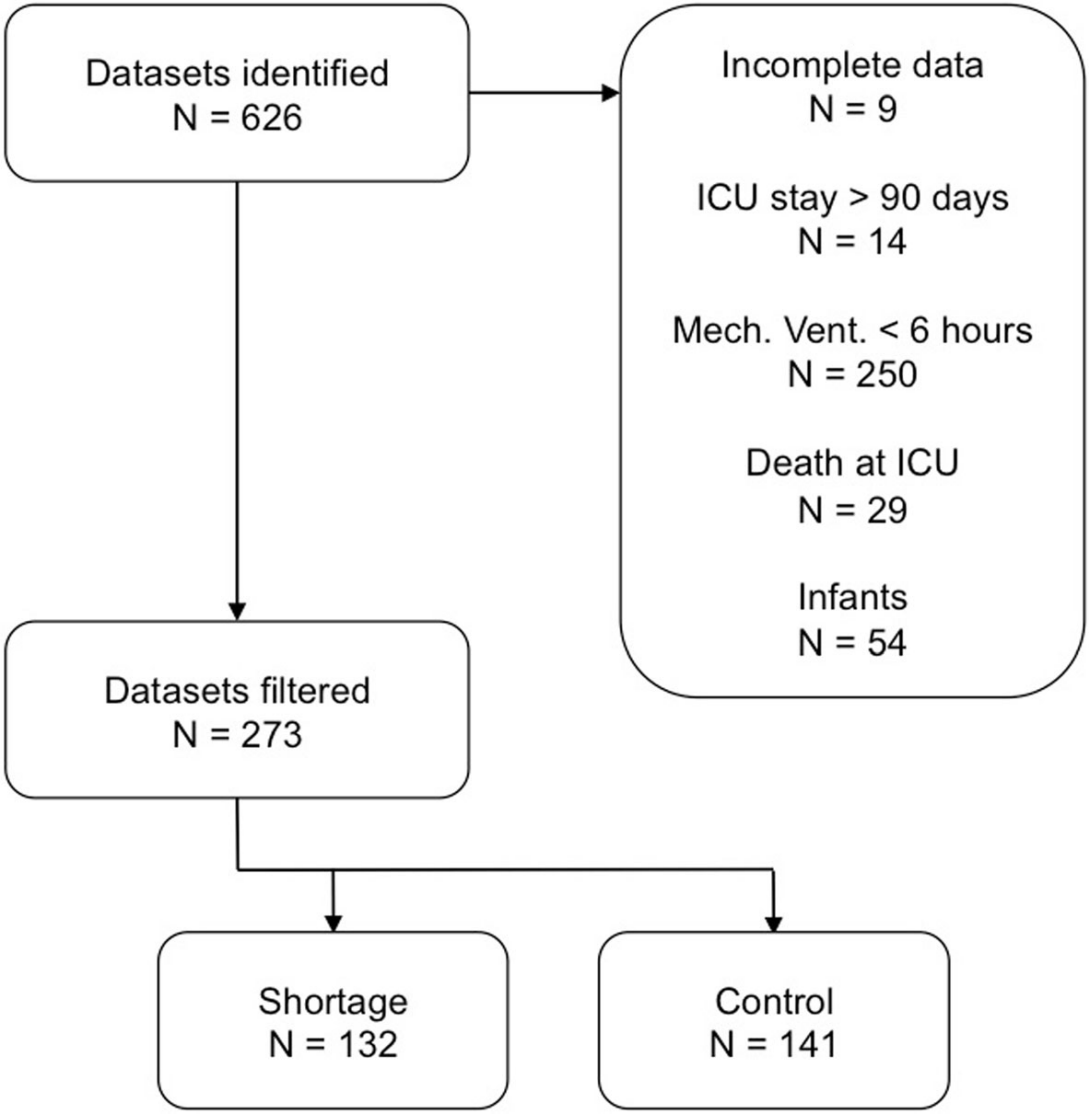
Flowchart of patient enrollment. Abbreviation: *ICU* intensive care unit

This retrospective study was conducted at three perioperative eight-bed ICUs of the General Hospital of Vienna. In the remifentanil shortage group, all patients were included when admitted to the ICU during the supply disruption between July 1, 2016 and September 30, 2016. As a control group, patients were included when admitted during the same period in 2017 to reduce confounding of seasonal variation. Patients with a medical reason for admission were included as well. Both elective and emergency patients were included. Exclusion criteria were incomplete data sets, ICU stay of more than 90 days, mechanical ventilation of below 6 hours, death in the ICU, and age of less than 18 years (Fig. 1). Sedoanalgesia was adapted by a bedside nurse in accordance with the institution’s standard with utilization of the Richmond Agitation-Sedation Scale (RASS) and the Numeric Rating Scale (NRS).

The primary outcome parameter was the duration of invasive mechanical ventilation, during which patients exhibited a secured airway (endotracheal tube or tracheal cannula) and underwent controlled or assisted ventilation. Termination of mechanical ventilation was defined as extubation in patients with endotracheal tube or as the time point without applied positive end-expiratory pressure (PEEP) or driving pressure in patients who underwent tracheostomy. Confounding variables for the primary outcome were age, sex, body mass index, admission Simplified Acute Physiology Score III (SAPS III), maximum of Sequential/Sepsis-related Organ Failure Assessment (SOFA) score, airway (primary tracheostomy: elective, operative tracheostomy with surgical indication; secondary tracheostomy: dilative tracheostomy due to prolonged mechanical ventilation), chronic obstructive pulmonary disease (COPD), intracranial operation or traumatic brain injury, coma, and delirium. Secondary outcome parameters were length of ICU stay, duration of non-invasive ventilation, length of hospital stay, occurrence of pneumonia as defined by the Infectious Diseases Society of America [12], and sepsis as defined by the sepsis consensus criteria [13]. Data were acquired by using the institutional database (IntelliSpace Critical Care and Anaesthesia, Philips, Eindhoven, The Netherlands).

### Statistics

For comparison of metric outcome parameters (ventilation days, length of ICU stay, duration of non-invasive ventilation, and length of hospital stay) between the two study groups, Poisson count regression models were computed with group (remifentanil shortage group and control group) as the main factor. For the primary outcome, confounding variables were computed. Dichotomous outcome parameters (pneumonia and sepsis) were analyzed with a logistic regression model. The significance level was split (alpha splitting) to compensate for multiple testing. Thus, a level of 0.02 was used for the primary and 0.006 for secondary outcome parameters. Frequencies of categories were compared between both groups by using Pearson’s chi-squared tests. In general, a two-tailed *P* value of below 0.05 was used as statistical level of significance. Data are given as mean ± standard deviation. All data were calculated with R Statistical software.

## Results

**Fig. 2:**
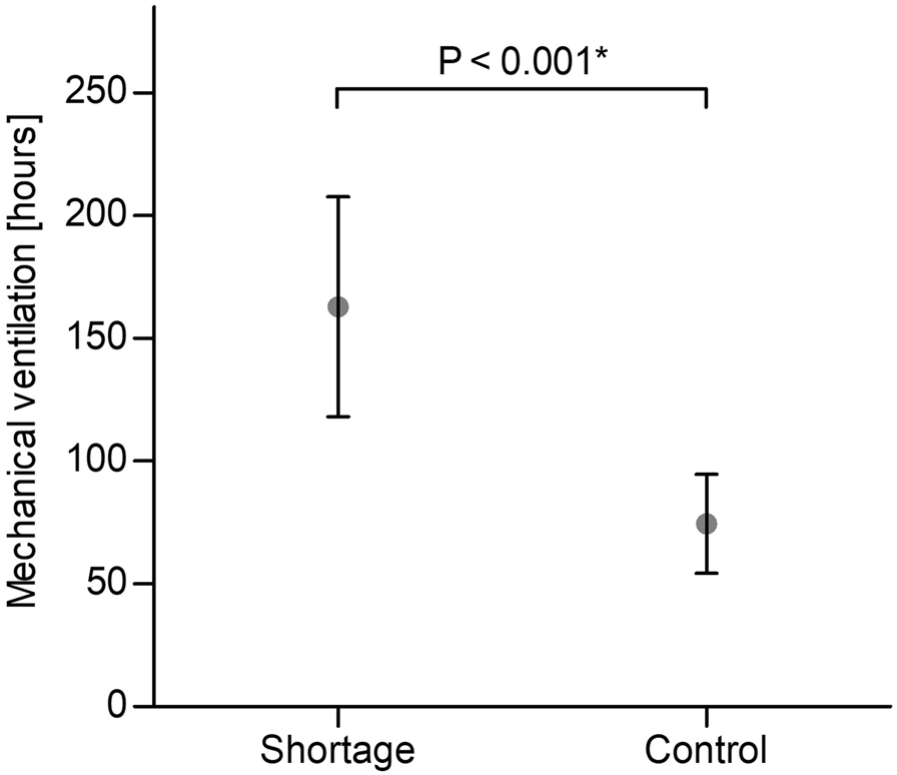
Primary outcome parameter. Dot and whiskers indicate mean with 95% confidence interval. Asterisk indicates significance

**Fig. 3:**
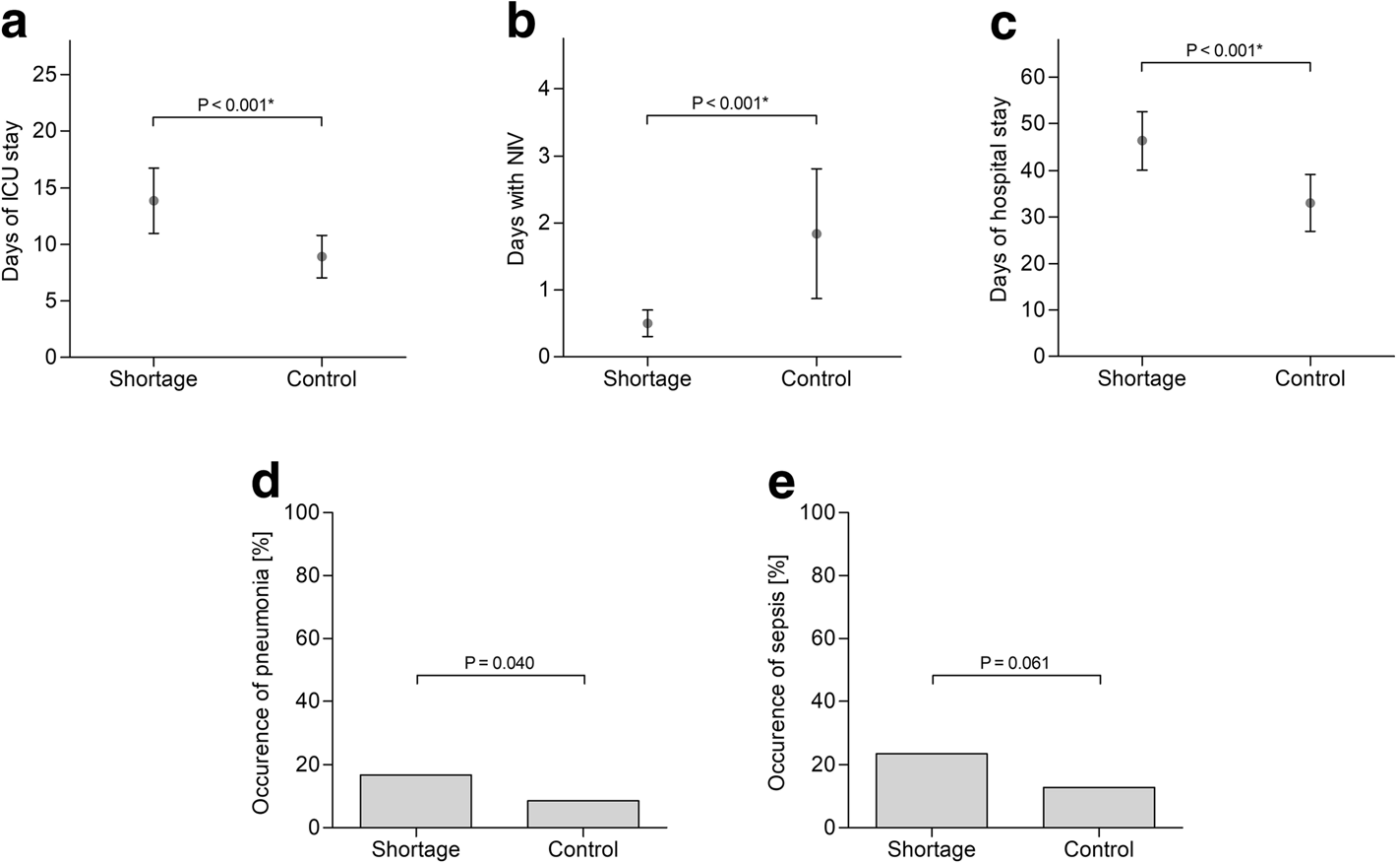
Secondary outcome parameters. Dots and whiskers indicate mean with 95% confidence interval. Asterisk indicates significance. **a** Length of intensive care unit (ICU) stay. **b** Days with non-invasive ventilation (NIV). **c** Length of hospital stay. **d** Pneumonia occurrence. **e** Sepsis occurrence

Data on the primary/secondary outcome parameter and confounding variables are listed in Table 1 and depicted in Figs. 2 and 3. The Poisson count regression model on duration of invasive mechanical ventilation illustrates a significant difference (*P* <0.001, Table 2). Age, sex, body mass index, admission SAPS III, maximum of SOFA score, tracheostomy, COPD, intracranial operation or traumatic brain injury, and delirium were significant confounders of ventilation hours. All secondary outcome parameters except pneumonia and sepsis were significantly increased in the remifentanil shortage group.

**Table 1:** Descriptive statistics of primary/secondary outcome parameters and confounding variables

Variable	Shortage (n = 132)	Control (n = 141)	*P* value
Primary outcome parameter			
Mechanical ventilation, hours	35 (14–211)	23 (13–66)	
Secondary outcome parameters			
Length of ICU stay, days	7 (3–17)	5 (3–10)	
Days with non-invasive ventilation	0 (0–0)	0 (0–1)	
Length of hospital stay, days	37 (17–63)	19 (12–41)	
Pneumonia occurrence, no.	31 (24)	18 (13)	
Sepsis occurrence, no.	22 (17)	12 (9)	
Confounding variables			
Age, years	58 (49–71)	58 (45–69)	0.720^a^
Sex, female	53 (40)	67 (48)	0.270^b^
Body mass index, kg m^−2^	25 (22–30)	24 (22–28)	0.153^a^
SAPS III at ICU admission	47 (40–57)	42 (34–50)	<0.001^c^
Maximum of SOFA score	8 (5–11)	7 (5–10)	0.600^c^
Airway			0.004^b^
Endotracheal tube	90 (68)	120 (85)	
Primary tracheostomy	17 (13)	8 (6)	
Secondary tracheostomy	25 (19)	13 (9)	
COPD	18 (14)	21 (15)	0.902^b^
Intracranial operation or TBI	19 (14)	22 (15)	0.913^b^
Coma	7 (5)	4 (3)	0.467^b^
Delirium	21 (16)	24 (17)	0.933^b^

Abbreviations: *COPD* chronic obstructive pulmonary disease, *ICU* intensive care unit, *SAPS III* Simplified Acute Physiology Score III, *SOFA* Sequential/Sepsis-related Organ Failure Assessment, *TBI* traumatic brain injury

Data are given as median with 25th and 75th percentile or absolute count with percentage

Statistical methods: ^a^Welch two-sample *t* test, ^b^Pearson’s chi-squared test with Yates’s continuity correction, ^c^Wilcoxon rank-sum test

**Table 2:** Regression models of outcome parameters

Model	Coefficient	RR	95% CI	Pr(>|z|)
Primary outcome: Invasive mechanical ventilation hours^a^				
Group only	Intercept	74.46	73.05–75.89	<0.001*
	Shortage group	2.19	2.14–2.24	<0.001*
Confounder	Intercept	20.89	19.39–22.49	<0.001*
	Shortage group	1.47	1.43–1.51	<0.001*
	Age	0.99	0.99–0.99	<0.001*
	Sex, female	1.08	1.06–1.11	<0.001*
	BMI^§^	1.019	1.016 -1.021	<0.001*
	SAPS III^§^	0.996	0.995–0.997	<0.001*
	SOFA_max_	1.114	1.109–1.118	<0.001*
	Prim. tracheostomy	1.18	1.11–1.26	<0.001*
	Sec. tracheostomy	5.68	5.52–5.84	<0.001*
	COPD	0.85	0.81–0.88	<0.001*
	Intracranial OP/TBI	1.61	1.56–1.66	<0.001*
	Coma	0.99	0.95–1.03	0.522
	Delirium	1.18	1.13–1.19	<0.001*
Secondary outcome: Length of ICU stay, days^a^				
Group only	Intercept	8.93	8.45–9.43	<0.001*
	Shortage group	1.55	1.45–1.67	<0.001*
Secondary outcome: Days with non-invasive ventilation, days^a^				
Group only	Intercept	1.84	1.62–2.08	<0.001*
	Shortage group	0.27	0.21–0.35	<0.001*
Secondary outcome: Length of hospital stay, days^a^				
Group only	Intercept	33.04	32.10–33.99	<0.001*
	Shortage group	1.40	1.35–1.46	<0.001*
Secondary outcome: Occurrence of pneumonia^b^				
Group only	Intercept	0.13	0.08–0.20	<0.001*
	Shortage group	1.84	1.04–3.35	0.040
Secondary outcome: Occurrence of sepsis^b^				
Group only	Intercept	0.09	0.05–0.14	<0.001*
	Shortage group	1.96	0.99–4.09	0.061

Abbreviations: *BMI* body mass index, *CI* confidence interval, *COPD* chronic obstructive pulmonary disease, *ICU* intensive care unit, *OP/TBI* intracranial operation/traumatic brain injury, *RR* risk ratio, *SAPS III* Simplified Acute Physiology Score III, *SOFA* Sequential/Sepsis-related Organ Failure Assessment

Statistical methods: ^a^Poisson count regression model. ^b^Logistic regression model. *Significant

^§^For clearer readability, the third decimal place was added

During the supply shortage, 36 patients (27%) received remifentanil in the ICU or during operations prior to ICU admission, contrary to 129 patients (92%) during the control period. Other continuously administrated opioids included fentanyl, sufentanil, morphine, and hydromorphone. The administration of all opioids but remifentanil was increased in the shortage group: Fentanyl was used in 89 (versus 55), sufentanil in 36 (versus 4), morphine in 22 (versus 1), and hydromorphone in 4 (versus 0) patients (Table 3). No significant difference was found in continuously infused sedative agents, which included propofol, dexmedetomidine, clonidine, ketamine, and midazolam (Table 4). The use of neuromuscular blocking agents did not differ significantly (*P* = 0.600).

**Table 3:** Dosages of opioids

Agent	Shortage	Control	*P* value
Remifentanil			
Used in x patients	36 (27)	129 (92)	<0.001^a^
Cum. dosage, mg	26.3 (8.8–90.3)	11.3 (4.5–29.1)	0.011^b^
Avg. dosage, μg kg^− 1^ min^− 1^	0.10 (0.01–0.15)	0.10 (0.07–0.16)	0.975^b^
Fentanyl			
Used in x patients	89 (67)	55 (39)	<0.001^a^
Cum. dosage, μg	1400 (800–2040)	1130 (910–1400)	0.066^b^
Avg. dosage, μg kg^−1^ h^− 1^	175 (111–232)	193 (150–150)	0.066^b^
Sufentanil			
Used in x patients	36 (27)	4 (3)	<0.001^a^
Cum. dosage, mg	3.9 (1.2–16.8)	16.9 (4.0–46.4)	0.443^b^
Avg. dosage, μg kg^−1^ h^− 1^	1.21 (0.79–1.79)	1.44 (0.54–1.71)	0.892^b^
Morphine			
Used in x patients	22 (17)	1 (1)	<0.001^a^
Cum. dosage, mg	76 (31–361)	15 (15–15)	0.131^b^
Avg. dosage, mg h^−1^	4.0 (2.4–5.1)	5.0 (5.0–5.0)	0.407^b^
Hydromorphone			
Used in x patients	4 (3)	0 (0)	0.115^a^
Cum. dosage, mg	24 (9–28)	–	–
Avg. dosage, mg h^−1^	0.28 (0.16–2.06)	–	–
Morphine equivalents pain management			
Cum. dosage, mg	23.6 (6.6–64.3)	27.1 (8.6–72.2)	0.350^b^

Data are given as absolute count with percentage or median with 25th and 75th percentile

Statistical methods: ^a^Pearson’s chi-squared test with Yates’s continuity correction, ^b^Wilcoxon rank-sum test

Cumulative dosages contain agents that were administered during intensive care unit admission and intraoperatively. Daily dosages are given per time or per body weight and time. “Morphine equivalents pain management” contains the m. e. of every additional opioid for pain management (intravenous bolus, oral opioids, and transdermal applications).

**Table 4:** Dosages of sedatives

Agent	Shortage	Control	*P* value
Propofol			
Used in x patients	132 (100)	139 (99)	0.507^a^
Cum. dosage, mg	3265 (1323–8869)	3353 (1230–7958)	0.535^b^
Avg. dosage, mg kg^− 1^ h^− 1^	1.94 (1.40–2.78)	2.22 (1.49–2.99)	0.084^b^
Dexmedetomidine			
Used in x patients	25 (19)	18 (14)	0.218^a^
Cum. dosage, μg	4801 (1016–14,748)	4652 (1795–7378)	0.873^b^
Avg. dosage, μg kg^−1^ h^− 1^	0.68 (0.41–1.02)	0.90 (0.63–1.13)	0.076^b^
Clonidine			
Used in x patients	16 (12)	17 (12)	0.866^a^
Cum. dosage, μg	8160 (4692–18,868)	7092 (3225–9606)	0.460^b^
Avg. dosage, μg h^−1^	85 (58–139)	90 (74–107)	0.857^b^
Ketamine			
Used in x patients	19 (14)	12 (9)	0.180^a^
Cum. dosage, mg	5900 (940–11,800)	3367 (519–12,538)	0.626^b^
Avg. dosage, mg h^−1^	441 (124–1082)	299 (60–2203)	0.612^b^
Midazolam			
Used in x patients	8 (6)	4 (3)	0.316^a^
Cum. dosage, mg	616 (271–1315)	2028 (645–3620)	0.089^b^
Avg. dosage, mg h^−1^	8 (4–11)	11 (8–17)	0.148^b^

Data are given as absolute count with percentage or median with 25th and 75th percentile

Statistical methods: ^a^Pearson’s chi-squared test with Yates’s continuity correction, ^b^Wilcoxon rank-sum test

Cumulative dosages contain agents that were administered during intensive care unit admission and intraoperatively. Daily dosages are given per time or per body weight and time

Gas exchange parameters, such as worst partial pressure of oxygen/fraction of inspired oxygen (paO_2_/FiO_2_) ratio (*P* = 0.115), FiO_2_ at ICU admission (*P* = 0.487), and partial pressure of carbon dioxide (paCO_2_) at ICU admission (*P* = 0.273) were not different; in contrast, paO_2_ at ICU admission was higher in the control group (*P* = 0.009). Overall, a low incidence of acute respiratory distress syndrome was observed, which did not differ significantly (0.668).

Between the two study groups, no significant difference was evident in frequency of re-intubation (*P* = 0.300) or in the usage of an extracorporeal membrane oxygenation (*P* = 0.406). Surgical disciplines (*P* = 0.159) or medical admissions (*P* = 0.541) did not differ significantly. The quantity of emergency admissions was similar between the two groups (*P* = 0.926). Descriptive data on RASS and NRS scores demonstrate trends toward lower NRS and RASS scores during the shortage period (Additional file 1).

## Discussion

This study reveals a significant increased risk ratio of mechanical ventilation hours with more than a twofold increase during a period with limited access to remifentanil as compared with an equivalent period with a corresponding patient collective. Furthermore, we found a significant increase of ICU length of stay, length of non-invasive ventilation, and length of hospital stay in the remifentanil shortage group. Incidence of pneumonia and sepsis did not differ between the study periods. A greater number of patients were included in the control group because of the elongated ICU stay in the shortage group with equal bed capacities.

Whereas an eight-year-old meta-analysis failed to identify remifentanil as a sufficient hypnotic sedative agent and argued for remifentanil’s non-inferiority as compared with other opioids [14], subsequent studies confirmed the opioid’s value regarding duration of mechanical ventilation, length of ICU stay, and reduction of sedatives [9, 15, 16]. This study provides novel evidence that restricted access to remifentanil remarkably deteriorated clinical outcome. Despite numerous significant confounding variables, the increase of the main outcome parameter remained at a high level of significance. Our data signify a higher morbidity with subsequent longer hospitalization during the drug shortage. Owing to a prolonged weaning and potential benefits [17], patients were more likely to undergo a secondary tracheostomy in the ICU, which is an invasive procedure that may cause laryngotracheal injury [18]. Moreover, the procedure was a significant confounder of all secondary outcome parameters (Additional file 1). Such outcomes may result in detrimental cost consequences for hospital cost carriers [19].

In the control group, the vast majority of patients received remifentanil, which reflects the primarily administrated sedoanalgetic regime with remifentanil and propofol established at the study location. The regime was implemented in accordance with the guideline for the management of delirium, analgesia, and sedation of the German Society of Anaesthesiology and Intensive Care Medicine [20]. In the remifentanil shortage group, still more than a quarter of patients received remifentanil. Remainders of previous deliveries maintained the availability for conditions with a need of a narrow sedoanalgetic control, such as polytrauma, traumatic brain injury, or lung transplantation. However, the unexpected supply disruption of this agent led to a broader choice of several opioids with a greater context-sensitive half-time [21] as well as a tendency to less pain and deeper sedation. Presumably, the sustained sedation and respiratory depression due to opioid hangover were responsible for a delayed appearance of sufficient extubation criteria. Given the concomitant disorientation or agitation in these patients, an additional or unfavorably timed administration of sedatives augments neurological alterations. Delays of an efficient respiratory weaning can occur, as can irregular sleep, decelerated mobilization, and deterioration of patient’s communication [22]. Interestingly, patients during the shortage had fewer days of non-invasive ventilation. This effect may occur because of a prolonged “saver” approach with a secured airway and therefore applied mechanical ventilation. The sufentanil usage was more common in the shortage group, which has been correlated with a greater dispersal of target sedation scales and administration of sedatives as compared with remifentanil [15].

The predominant handling with remifentanil at the study location may minimize the proficiency with other continuously infused opioids. Subsequently, physicians were possibly less experienced with these substances. Recent literature reports clinical effects of drug shortages concerning anesthesia and intensive care medicine. Such supply shortages provoked impacts not only on sedoanalgetic regimes [23, 24] but also on hemodynamics and mortality [25]. Inadequate accessibility of familiar medications forces physicians to switch to alternative treatment procedures. This may result in uncertainty or an inadequate dosage adjustment.

Occurrences of pneumonia and sepsis were not statistically different but appear with risk ratios similar or even higher as compared with the significant outcome parameters. The low number of cases as well as the compensated level of significance may contribute to these results. Supposedly, an extended shortage may have led to more cases and more distinct *P* values.

SAPS III was greater in the shortage group, whereas maxima of SOFA scores were equal between the study groups. Both scores are calculated digitally by the patient data management system, which uses the worst parameters within a 24-h period. In the present study, SAPS III was assessed only once at ICU admission. SOFA scores were screened daily and may provide a more precise resolution regarding severity of acute illness. Furthermore, the Poisson count regression model on mechanical ventilation includes SAPS III as confounder, where the outcome parameter remained significant. Providing a mortality predicting system, the role of SAPS III in this study may be attenuated since death in the ICU was an exclusion criterion.

Death in the ICU was determined as an exclusion criterion for several rationales. This study’s aim, with regard to the primary outcome, was to investigate the influence on ICU survivors who are able to be weaned from an invasive mechanical ventilation. Alterations due to the drug shortage were assumed to occur during the respiratory weaning. Furthermore, patients who underwent comfort terminal care, in which mechanical ventilation and opioid administration are managed with different therapeutic goals, are excluded.

Patients with an ICU stay of greater than 90 days were excluded since this time would exceed the possible time of exposure. Patients with a long ICU stay are at risk of being exposed to a greater proportion to a non-shortage period. However, excluding non-survivors and patients with a prolonged ICU stay remains a limitation of this study.

Predominantly, patients in this study were admitted postoperatively; only 6% were admitted for medical reasons. Although medically admitted patients trend to longer mechanical ventilation (median in shortage group: 296 h, control group: 160 h), the number of cases is low and our findings may not apply to a medical ICU population.

Experience of physicians and grade of familiarity with opioid administration were not assessed in this study. Staff fluctuation and the narrow observation period are limiting the analysis of these factors. Possibly, frequent handling with other substances may have led to a subsequent reduction of ventilation time.

Since this study extracted data from ICUs at a tertiary care hospital, data from three ICUs were included. The ICUs receive the same patient collectives, provide the same medical management, and are staffed predominantly with similar physicians. Nonetheless, this study is a single-center study that is not able to extrapolate the findings on nationwide outcomes. Research with nationwide data concerning such broad and persistent drug shortages is necessary to detect potential disturbances of medical care.

## Conclusions

During a shortage of remifentanil availability, patients underwent prolonged mechanical ventilation with an increased incidence of associated complications. The findings of this study support the necessity of outcome monitoring during drug shortages, particularly of ICU outcomes.

## Additional file

Additional file 1: Supplementary material: Descriptive statistics and figures, regression models of secondary parameters and confounders. (DOCX 968 kb)

## Abbreviations

COPD: Chronic obstructive pulmonary disease
FiO_2_: Partial pressure of oxygen
ICU: Intensive care unit
NRS: Numeric Rating Scale
PaO_2_: Fraction of inspired oxygen
RASS: Richmond Agitation-Sedation Scale
SAPS III: Simplified Acute Physiology Score III
SOFA: Sequential/Sepsis-related Organ Failure Assessment

## References

[1] Patel SB, Kress JP (2012). Sedation and analgesia in the mechanically ventilated patient. Am J Respir Crit Care Med..

[2] Roberts DJ, Haroon B, Hall RI (2012). Sedation for critically ill or injured adults in the intensive care unit: a shifting paradigm. Drugs.

[3] Celis-Rodriguez E, Birchenall C, de la Cal MA, Castorena Arellano G, Hernandez A, Ceraso D (2013). Clinical practice guidelines for evidence-based management of sedoanalgesia in critically ill adult patients. Med Int..

[4] Romsing J, Moiniche S, Mathiesen O, Dahl JB (2005). Reduction of opioid-related adverse events using opioid-sparing analgesia with COX-2 inhibitors lacks documentation: a systematic review. Acta Anaesthesiol Scand..

[5] Lewis SC, Li L, Murphy MV, Klompas M, CDC Prevention Epicenters (2014). Risk factors for ventilator-associated events: a case-control multivariable analysis. Crit Care Med..

[6] Bouadma L, Sonneville R, Garrouste-Orgeas M, Darmon M, Souweine B, Voiriot G (2015). Ventilator-associated events: prevalence, outcome, and relationship with ventilator-associated pneumonia. Crit Care Med..

[7] McGrane S, Pandharipande PP (2012). Sedation in the intensive care unit. Minerva Anestesiol..

[8] Rosow C (1993). Remifentanil: a unique opioid analgesic. Anesthesiology.

[9] Lebherz-Eichinger D, Tudor B, Krenn CG, Roth GA, Seemann R (2016). Impact of different sedation protocols and perioperative procedures on patients admitted to the intensive care unit after maxillofacial tumor surgery of the lower jaw: A retrospective study. J Craniomaxillofac Surg..

[10] Rozendaal FW, Spronk PE, Snellen FF, Schoen A, van Zanten AR, Foudraine NA (2009). Remifentanil-propofol analgo-sedation shortens duration of ventilation and length of ICU stay compared to a conventional regimen: a centre randomised, cross-over, open-label study in the Netherlands. Int Care Med..

[11] Dahaba AA, Grabner T, Rehak PH, List WF, Metzler H (2004). Remifentanil versus morphine analgesia and sedation for mechanically ventilated critically ill patients: a randomized double blind study. Anesthesiology.

[12] Kalil AC, Metersky ML, Klompas M, Muscedere J, Sweeney DA, Palmer LB (2016). Management of Adults With Hospital-acquired and Ventilator-associated Pneumonia: 2016 Clinical Practice Guidelines by the Infectious Diseases Society of America and the American Thoracic Society. Clin Infect Dis..

[13] Rhodes A, Evans LE, Alhazzani W, Levy MM, Antonelli M, Ferrer R (2017). Surviving Sepsis Campaign: International Guidelines for Management of Sepsis and Septic Shock: 2016. Int Care Med..

[14] Tan JA, Ho KM (2009). Use of remifentanil as a sedative agent in critically ill adult patients: a meta-analysis. Anaesthesia.

[15] Futier E, Chanques G, Cayot Constantin S, Vernis L, Barres A, Guerin R (2012). Influence of opioid choice on mechanical ventilation duration and ICU length of stay. Minerva Anestesiol..

[16] Welzing L, Oberthuer A, Junghaenel S, Harnischmacher U, Stutzer H, Roth B (2012). Remifentanil/midazolam versus fentanyl/midazolam for analgesia and sedation of mechanically ventilated neonates and young infants: a randomized controlled trial. Int Care Med..

[17] Andriolo BN, Andriolo RB, Saconato H, Atallah AN, Valente O (2015). Early versus late tracheostomy for critically ill patients. Cochrane Database Syst Rev..

[18] Liu CC, Livingstone D, Dixon E, Dort JC (2015). Early versus late tracheostomy: a systematic review and meta-analysis. Otolaryngol Head Neck Surg..

[19] Al MJ, Hakkaart L, Tan SS, Bakker J (2010). Cost-consequence analysis of remifentanil-based analgo-sedation vs. conventional analgesia and sedation for patients on mechanical ventilation in the Netherlands. Crit Care.

[20] Baron R, Binder A, Biniek R, Braune S, Buerkle H, DAS-Taskforce 2015 (2015). Evidence and consensus based guideline for the management of delirium, analgesia, and sedation in intensive care medicine. Revision 2015 (DAS-Guideline 2015) - short version. Ger Med Sci.

[21] Wilhelm W, Kreuer S (2008). The place for short-acting opioids: special emphasis on remifentanil. Crit Care.

[22] Vincent JL, Shehabi Y, Walsh TS, Pandharipande PP, Ball JA, Spronk P (2016). Comfort and patient-centred care without excessive sedation: the eCASH concept. Int Care Med..

[23] Roberts R, Ruthazer R, Chi A, Grover A, Newman M, Bhat S (2012). Impact of a national propofol shortage on duration of mechanical ventilation at an academic medical center. Crit Care Med..

[24] Thoma BN, Li J, McDaniel CM, Wordell CJ, Cavarocchi N, Pizzi LT (2014). Clinical and economic impact of substituting dexmedetomidine for propofol due to a US drug shortage: examination of coronary artery bypass graft patients at an urban medical centre. PharmacoEconomics.

[25] Vail E, Gershengorn HB, Hua M, Walkey AJ, Rubenfeld G, Wunsch H (2017). Association between US norepinephrine shortage and mortality among patients with septic shock. JAMA.

