# Supplementary material for: Impact of a remifentanil supply shortage on mechanical ventilation in a tertiary care hospital: a retrospective comparison
Source: Crit Care. 2018 Oct 26;22:267. doi: 10.1186/s13054-018-2198-3 (PMC6204001; doi:10.1186/s13054-018-2198-3)
Supplement: Supplementary file 1 — Supplementary material: Descriptive statistics and figures, regression models of secondary parameters and confounders. (DOCX 968 kb) [file 13054_2018_2198_MOESM1_ESM.docx]

Supplementary material

**Article:**

Impact of a remifentanil supply shortage on mechanical ventilation in a tertiary care hospital – a retrospective comparison

**Journal:**

Critical Care

**Authors:**

Daniel A. Klaus^1,2^, Albert M. de Bettignies^1,2^, Rudolf Seemann^3^, Claus G. Krenn^1,2^, Georg A. Roth^1,2^

**Affiliations:**

^1^Department of Anesthesiology, General Intensive Care and Pain Medicine, Medical University of Vienna, Vienna, Austria

^2^RAIC Laboratory 13C1, Medical University of Vienna, Vienna, Austria

^3^Department of Craniomaxillofacial and Oral Surgery, Medical University of Vienna, Vienna, Austria

**Corresponding author:**

Daniel A. Klaus; Mail: daniel.klaus@meduniwien.ac.at

**Content:**

Descriptive statistics and figures

Regression models of secondary parameters and confounders

# Descriptive data and figures

## Distribution of disciplines

Figure S1.2. Surgical disciplines and medical admission reasons over study groups (absolute counts)


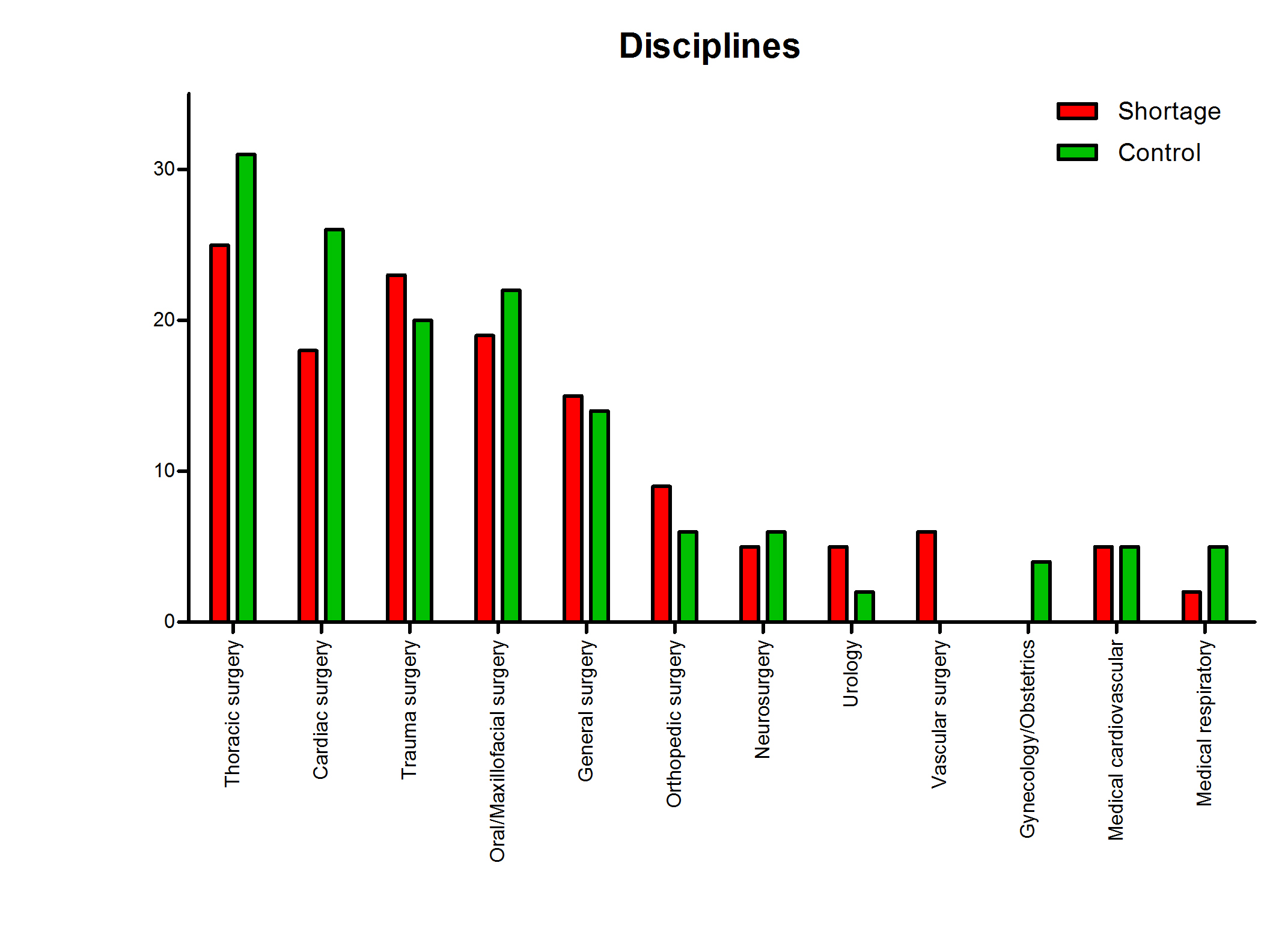


Table S1.1. Absolute counts of disciplines

|  | Thoracic surgery | Cardiac surgery | Trauma surgery | Oral / Maxillofacial surgery | General surgery | Orthopedic surgery | Neurosurgery |
| --- | --- | --- | --- | --- | --- | --- | --- |
| Shortage | 25 | 18 | 23 | 19 | 15 | 9 | 5 |
| Control | 31 | 26 | 20 | 22 | 14 | 6 | 6 |
|  | Urology | Vascular surgery | Gynecology / Obs | Medical cardiovascular | Medical respiratory | Total |  |
| Shortage | 5 | 6 | 0 | 5 | 2 | 132 |  |
| Control | 2 | 0 | 4 | 5 | 5 | 141 |  |

## Admission types

|  | | Shortage  (n = 132) | | Control  (n = 141) | |
| --- | --- | --- | --- | --- | --- |
| Reason for admission |  | |  | | 0.541^a^ |
| Perioperative | 125 (95) | | 131 (93) | |  |
| Medical | 7 (5) | | 10 (7) | |  |
| Type of admission |  | |  | | 0.926^a^ |
| Emergency | 47 (36) | | 52 (37) | |  |
| Elective | 85 (64) | | 89 (63) | |  |
| Data are given as absolute count with percentage.  Statistical methods: ^a^Pearson’s Chi-squared test with Yates’ continuity correction. | | | | | |

## Gas exchange, proportion of days with mechanical ventilation, ARDS, use of neuromuscular blocking agents

Table S1.3. Gas exchange parameters, days with mechanical ventilation

|  | | Shortage  (n = 132) | | Control  (n = 141) | |
| --- | --- | --- | --- | --- | --- |
| Worst paO_2_/FiO_2_ ratio | 203 (129-286) | | 223 (141-317) | | 0.115^a^ |
| FiO_2_ at ICU admission [%] | 40 (40-50) | | 40 (35-50) | | 0.487^a^ |
| paO_2_ at ICU admission [mm HG] | 124 (93-151) | | 138 (106-170) | | 0.009^a^ |
| paCO_2_ at ICU admission [mm HG] | 41 (37-45) | | 39 (32-46) | | 0.273^a^ |
| Relation days with mechanical ventilation and length of ICU stay [days/days] | 0.50 (0.33 - 0.63) | | 0.36 (0.25 - 0.50) | | < 0.001^a^ |
| Incidence of ARDS | 6 (4.5) | | 4 (2.8) | | 0.668^b^ |
| Use of NMBAs | 22 (17) | | 28 (20) | | 0.600 ^b^ |
| Data are given as median with 25^th^ and 75^th^ percentile or as absolute count with percentage.  Statistical methods: ^a^Wilcoxon rank sum test; ^b^Pearson’s Chi-squared test with Yates’ continuity correction. | | | | | |

## Trends of Richmond Agitation Sedation Scale (RASS)

Sedoanalgesia was adapted by a bedside nurse according to institution’s standard with utilization of the Richmond Agitation Sedation Scale (RASS) and the Numeric Rating Scale (NRS). Both scores were assessed at least twice per day. RASS values were included, if mechanical ventilation (primary outcome) was applied.

Figures illustrate time course over the first 10 days. Dots and whiskers indicate mean with 95% confidence interval.


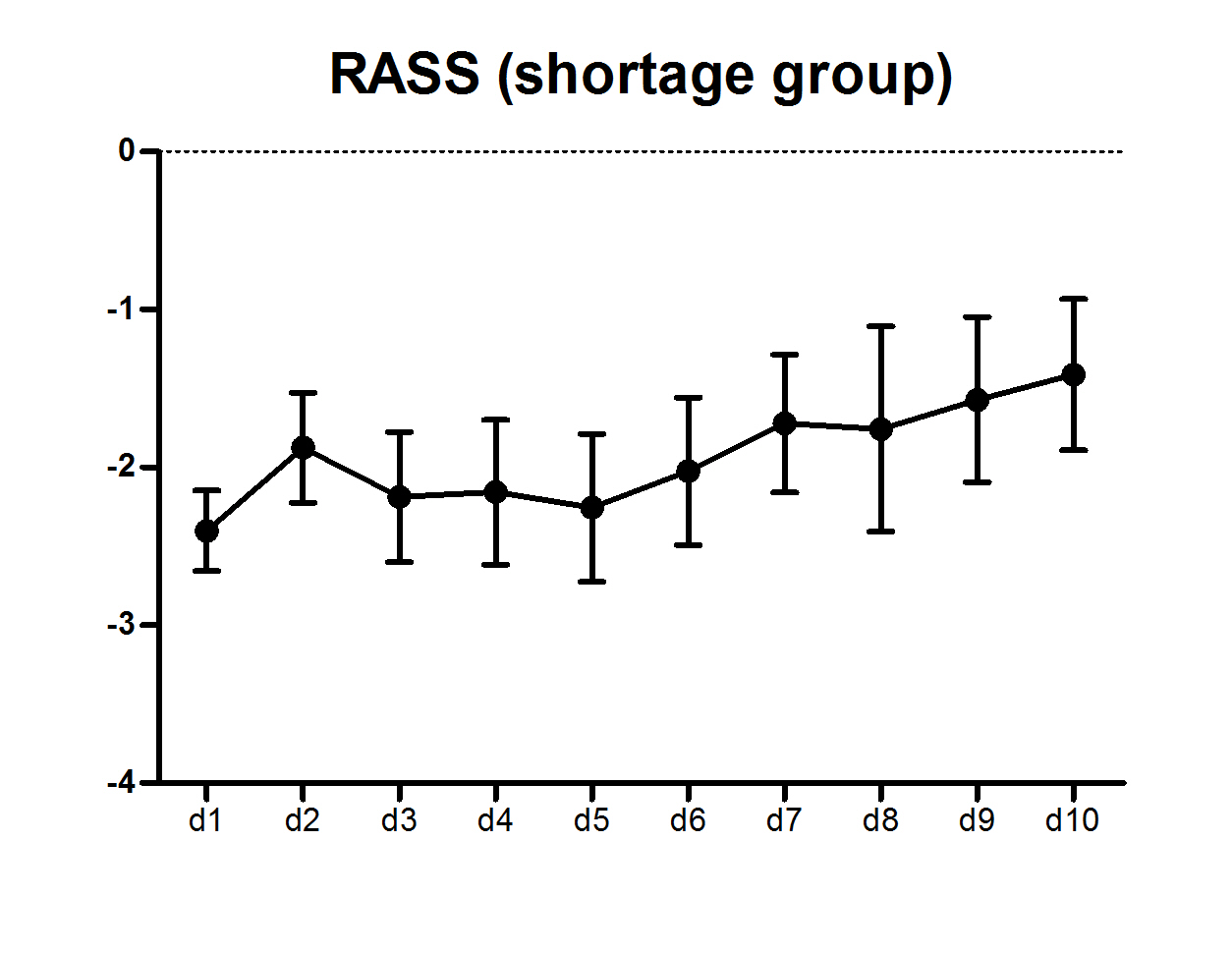

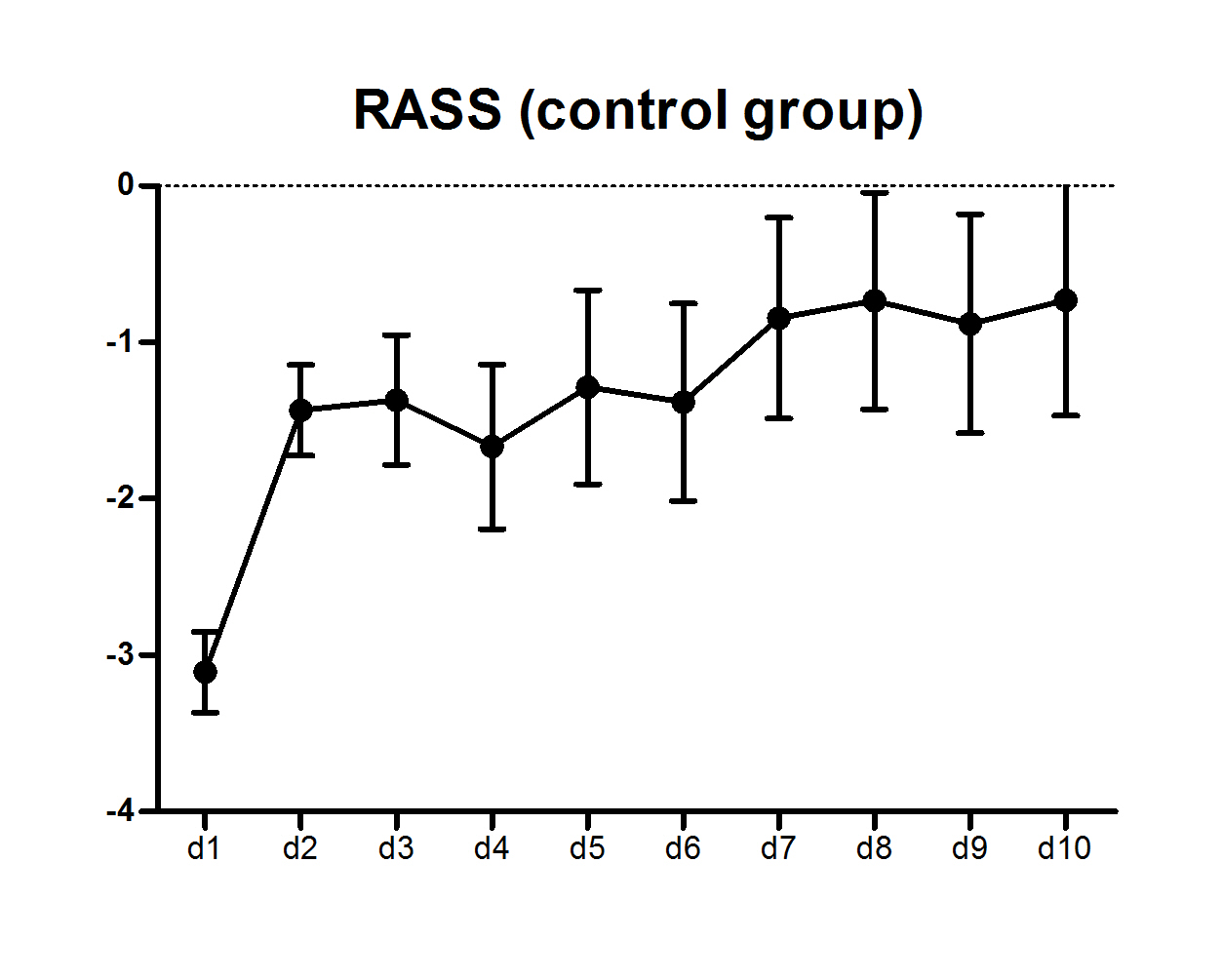


**Table 1.3.1. RASS scores**

|  | d1 | d2 | d3 | d4 | d5 | d6 | d7 | d8 | d9 | d10 |
| --- | --- | --- | --- | --- | --- | --- | --- | --- | --- | --- |
| Shortage | -2,4 ± 1,5 | -1,9 ± 1,7 | -2,2 ± 1,6 | -2,2 ± 1,6 | -2,3 ± 1,6 | -2,0 ± 1,5 | -1,7 ± 1,4 | -1,8 ± 1,9 | -1,6 ± 1,5 | -1,4 ± 1,4 |
| Control | -3,1 ± 1,5 | -1,4 ± 1,6 | -1,4 ± 1,7 | -1,7 ± 1,9 | -1,3 ± 2,0 | -1,4 ± 1,8 | -0,8 ± 1,8 | -0,7 ± 1,8 | -0,9 ± 1,7 | -0,7 ± 1,8 |
| Data are given as daily mean ± standard deviation. | | | | | | | | | | |

## Trends of Numerical Rating Scale for pain assessment (NRS)

Correspondent to RASS, NRS values were included, if mechanical ventilation (primary outcome) was applied. If NRS values was unable to evaluate (e.g. deep sedation), the Behavior Pain Scale (BPS) was used for pain assessment. In this case, values were converted to NRS equivalents.

Dots and whiskers indicate mean with 95% confidence interval.


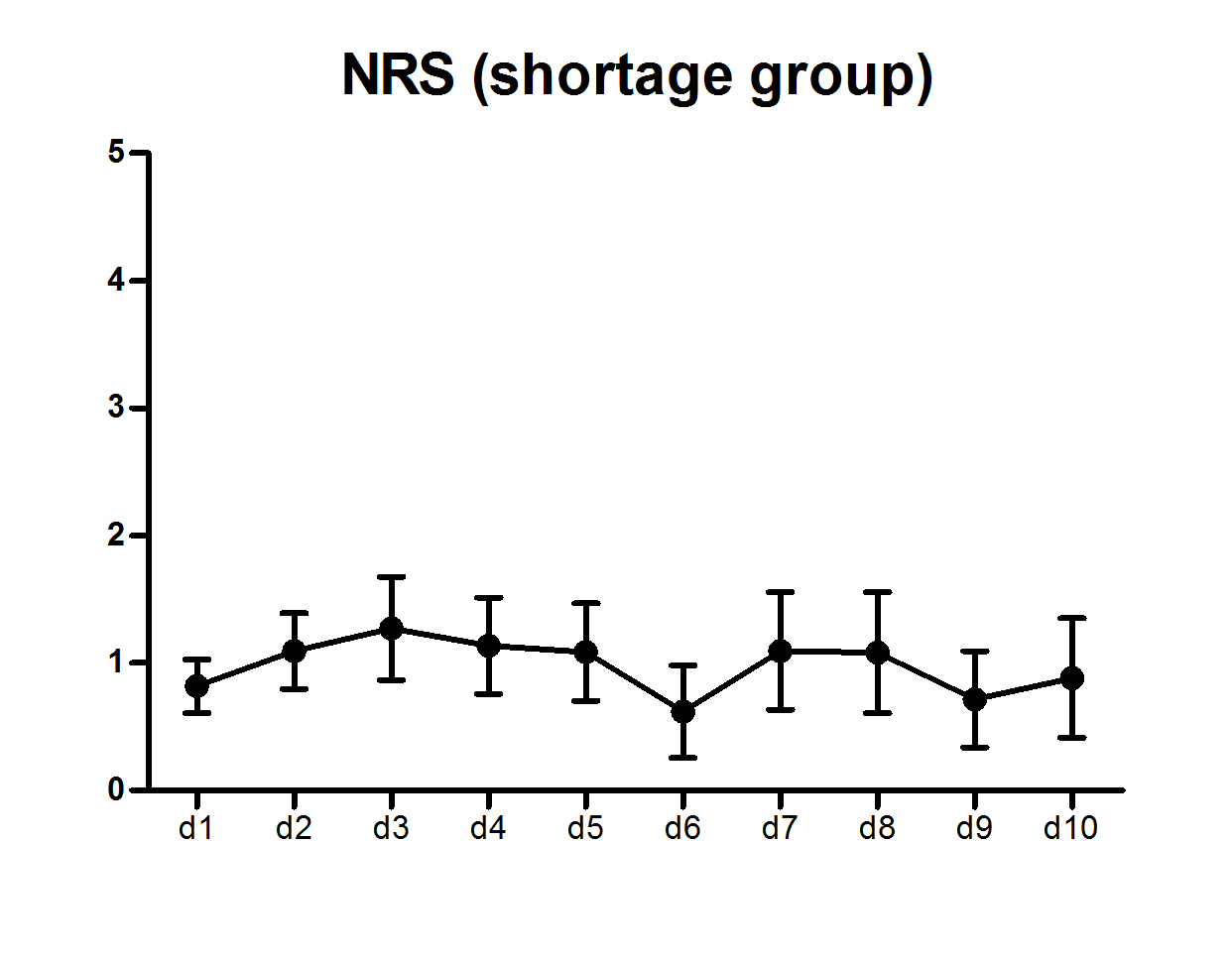

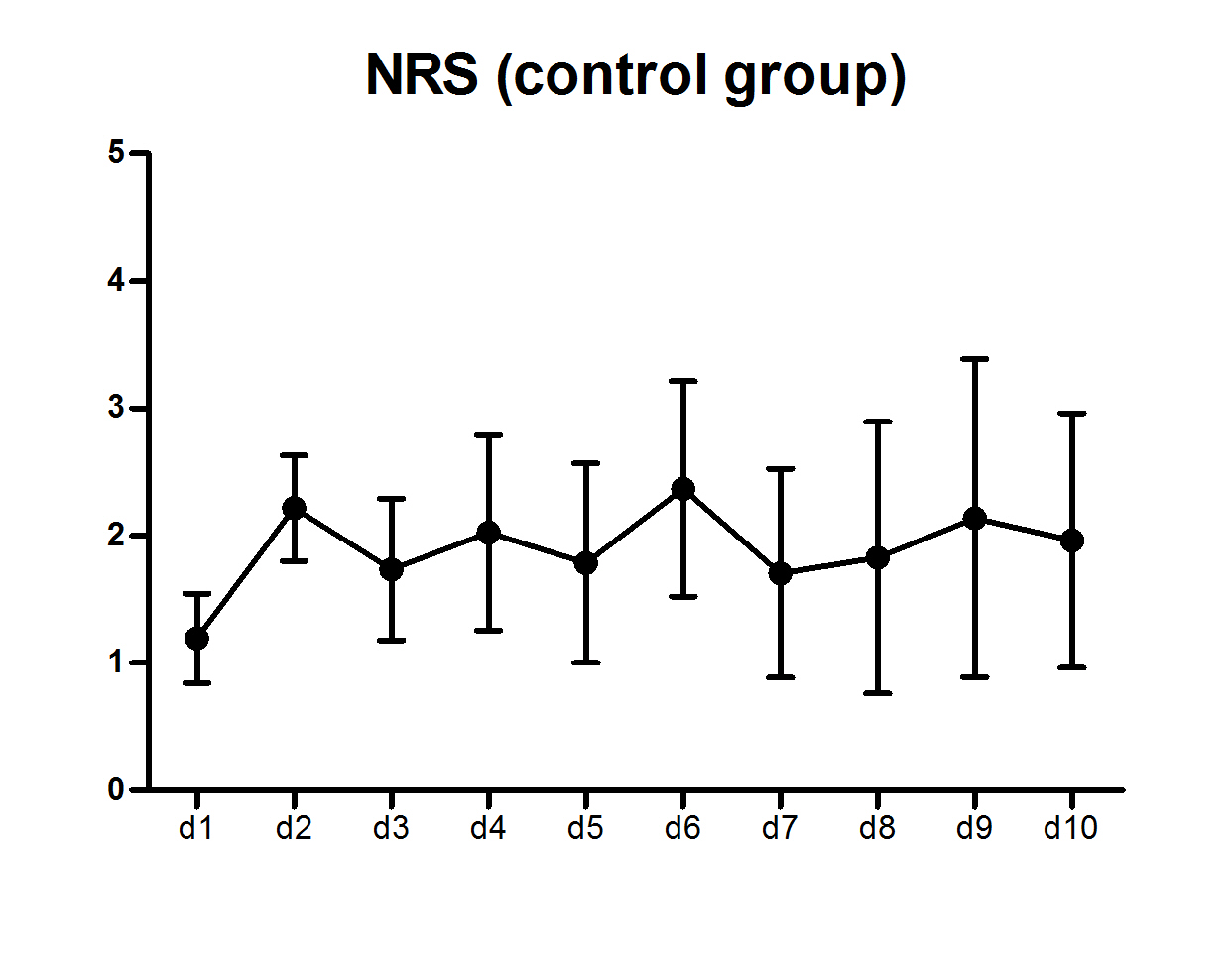


**Table 1.4.1 NRS scores**

|  | d1 | d2 | d3 | d4 | d5 | d6 | d7 | d8 | d9 | d10 |
| --- | --- | --- | --- | --- | --- | --- | --- | --- | --- | --- |
| Shortage | 0,8 ± 1,2 | 1,1 ± 1,5 | 1,3 ± 1,6 | 1,1 ± 1,3 | 1,1 ± 1,3 | 0,6 ± 1,2 | 1,1 ± 1,5 | 1,1 ± 1,4 | 0,7 ± 1,1 | 0,9 ± 1,3 |
| Control | 1,2 ± 2,0 | 2,2 ± 2,2 | 1,7 ± 2,1 | 2,0 ± 2,5 | 1,8 ± 2,3 | 2,4 ± 2,2 | 1,7 ± 2,0 | 1,8 ± 2,4 | 2,1 ± 2,8 | 2,0 ± 2,4 |
| Data are given as daily mean ± standard deviation. | | | | | | | | | | |

# Regression models of secondary parameters and confounders

Table S2.1. Poisson count regression model of length of ICU stay

| Model | Coefficient | RR | 95% CI | Pr(>\|z\|) |
| --- | --- | --- | --- | --- |
| Confounder | Intercept | 3.38 | 2.67 - 4.26 | <0.001 * |
|  | Shortage Group | 1.26 | 1.17 - 1.36 | <0.001 * |
|  | Age | 0.993 | 0.991 - 0.996 | <0.001 * |
|  | Sex | 1.15 | 1.07 - 1.25 | < 0.001 * |
|  | BMI | 1.01 | 1.00 - 1.02 | < 0.001 * |
|  | SAPS III | 0.997 | 0.994 - 1.000 | 0.044 * |
|  | SOFAmax | 1.11 | 1.10 - 1.12 | < 0.001 * |
|  | Prim. tracheostomy | 0.76 | 0.62 - 0.92 | 0.006 * |
|  | Sec. tracheostomy | 2.83 | 2.58 - 3.10 | < 0.001 * |
|  | COPD | 0.87 | 0.76 - 0.98 | 0.028 * |
|  | Intracranial OP / TBI | 1.47 | 1.34 - 1.62 | < 0.001 * |
|  | Coma | 0.93 | 0.79 - 1.08 | 0.334 |
|  | Delirium | 1.24 | 1.13 - 1.36 | < 0.001 * |
| RR: Risk ratio; CI: confidence interval. *Significant. | | | | |

Table S2.2. Poisson count regression model of days with non-invasive ventilation and confounders

| Model | Coefficient | RR | 95% CI | Pr(>\|z\|) |
| --- | --- | --- | --- | --- |
| Confounder | Intercept | 0.34 | 0.16 - 0.73 | 0.005 * |
|  | Shortage Group | 0.24 | 0.17 - 0.31 | <0.001 * |
|  | Age | 0.996 | 0.988 - 1.004 | 0.330 |
|  | Sex [female] | 1.14 | 0.88 - 1.48 | 0.321 |
|  | BMI | 0.99 | 0.97 - 1.01 | 0.285 |
|  | SAPS III | 0.99 | 0.98 - 1.00 | 0.071 |
|  | SOFAmax | 1.29 | 1.23 - 1.34 | < 0.001 * |
|  | Prim. tracheostomy | 0.54 | 0.19 - 1.22 | 0.187 |
|  | Sec. tracheostomy | 2.18 | 1.63 - 2.90 | < 0.001 * |
|  | COPD | 1.78 | 1.25 - 2.50 | < 0.001 * |
|  | Intracranial OP / TBI | 2.49 | 1.85 - 3.33 | < 0.001 * |
|  | Coma | 0.53 | 0.30 - 0.89 | 0.022 * |
|  | Delirium | 0.76 | 0.57 - 1.02 | 0.067 |
| RR: Risk ratio; CI: confidence interval. *Significant. | | | | |

Table S2.3. Poisson count regression model of length of hospital stay and confounders

| Model | Coefficient | RR | 95% CI | Pr(>\|z\|) |  |
| --- | --- | --- | --- | --- | --- |
| Confounder | Intercept | 18.82 | 16.56 - 21.39 | <0.001 * |  |
|  | Shortage Group | 1.24 | 1.19 - 1.29 | <0.001 * |  |
|  | Age | 0.997 | 0.995 - 0.998 | <0.001 * |  |
|  | Sex [female] | 1.19 | 1.14 - 1.24 | < 0.001 * |  |
|  | BMI | 0.997 | 0.994 - 1.001 | 0.159 |  |
|  | SAPS III | 1.005 | 1.003 - 1.006 | < 0.001 * |  |
|  | SOFAmax | 1.04 | 1.03 - 1.05 | < 0.001 * |  |
|  | Prim. tracheostomy | 1.70 | 1.59 - 1.81 | < 0.001 * |  |
|  | Sec. tracheostomy | 1.49 | 1.41 - 1.58 | < 0.001 * |  |
|  | COPD | 0.96 | 0.90 - 1.02 | 0.172 |  |
|  | Intracranial OP / TBI | 1.26 | 1.19 - 1.33 | < 0.001 * |  |
|  | Coma | 1.36 | 1.24 - 1.49 | < 0.001 * |  |
|  | Delirium | 1.39 | 1.32 - 1.47 | < 0.001 * |  |
| RR: Risk ratio; CI: confidence interval. *Significant. | | | | | |

Table S2.4. Logistic regression model of pneumonia occurrence and confounders

| Model | Coefficient | RR | 95% CI | Pr(>\|z\|) |
| --- | --- | --- | --- | --- |
| Confounder | Intercept | 0.02 | 0.00 - 0.12 | <0.001 * |
|  | Shortage Group | 1.22 | 0.65 - 2.30 | 0.542 |
|  | Age | 1.00 | 0.98 - 1.01 | 0.606 |
|  | Sex [female] | 0.85 | 0.41 - 1.68 | 0.640 |
|  | BMI | 1.04 | 0.99 - 1.09 | 0.148 |
|  | SAPS III | 1.00 | 0.98 - 1.03 | 0.728 |
|  | SOFAmax | 1.07 | 0.96 - 1.18 | 0.211 |
|  | Prim. tracheostomy | 1.05 | 0.16 - 3.88 | 0.950 |
|  | Sec. tracheostomy | 4.53 | 2.18 - 9.46 | < 0.001 * |
|  | COPD | 0.99 | 0.35 - 2.37 | 0.978 |
|  | Intracranial OP / TBI | 1.16 | 0.49 - 2.52 | 0.726 |
|  | Coma | 0.87 | 0.25 - 2.64 | 0.818 |
|  | Delirium | 1.93 | 0.97 - 3.82 | 0.059 |
| RR: Risk ratio; CI: confidence interval. *Significant. | | | | |

Table S2.5. Logistic regression model of sepsis occurrence and confounders

| Model | Coefficient | RR | 95% CI | Pr(>\|z\|) |
| --- | --- | --- | --- | --- |
| Confounder | Intercept | 0.008 | 0.001 - 0.057 | <0.001 * |
|  | Shortage Group | 1.22 | 0.57 - 2.69 | 0.619 |
|  | Age | 0.99 | 0.96 - 1.01 | 0.368 |
|  | Sex [female] | 0.98 | 0.42 - 2.23 | 0.969 |
|  | BMI | 1.04 | 0.97 - 1.10 | 0.258 |
|  | SAPS III | 1.01 | 0.99 - 1.04 | 0.345 |
|  | SOFAmax | 1.15 | 1.01 - 1.31 | 0.035 * |
|  | Prim. tracheostomy | 2.38 | 0.51 - 8.23 | 0.205 |
|  | Sec. tracheostomy | 3.95 | 1.62 - 9.60 | 0.002 * |
|  | COPD | 1.54 | 0.53 - 3.88 | 0.386 |
|  | Intracranial OP / TBI | 1.00 | 0.27 - 2.96 | 0.999 |
|  | Coma | 0.35 | 0.05 - 1.46 | 0.203 |
|  | Delirium | 0.87 | 0.36 - 1.95 | 0.742 |
| RR: Risk ratio; CI: confidence interval. *Significant. | | | | |
